# Atrial and ventricular strain using cardiovascular magnetic resonance in the prediction of outcomes of pericarditis patients: a pilot study

**DOI:** 10.1007/s00330-024-10677-9

**Published:** 2024-03-11

**Authors:** Riccardo Cau, Francesco Pisu, Giuseppe Muscogiuri, Sandro Sironi, Jasjit S. Suri, Gianluca Pontone, Rodrigo Salgado, Luca Saba

**Affiliations:** 1 Department of Radiology, Azienda Ospedaliero Universitaria (A.O.U.), di Cagliari - Polo di Monserrato s.s. 554, Monserrato, 09045 Cagliari, Italy; 2 School of Medicine and Surgery, University of Milano-Bicocca, Milan, Italy; 3 Department of Radiology, IRCCS Istituto Auxologico Italiano, San Luca Hospital, Milan, Italy; 4 Stroke Monitoring and Diagnostic Division, AtheroPoint™, Roseville, CA USA; 5 Department of Perioperative Cardiology and Cardiovascular Imaging, Centro Cardiologico Monzino IRCCCS, Milan, Italy; 6 Department of Biomedical, Surgical and Dental Sciences, University of Milan, Milan, Italy; 7 Universitair Ziekenhuis Antwerpen, Edegem, Belgium

**Keywords:** Pericarditis, Magnetic resonance imaging, Prognosis, Strain

## Abstract

**Objective:**

Our study aimed to explore with cardiovascular magnetic resonance (CMR) the impact of left atrial (LA) and left ventricular (LV) myocardial strain in patients with acute pericarditis and to investigate their possible prognostic significance in adverse outcomes.

**Method:**

This retrospective study performed CMR scans in 36 consecutive patients with acute pericarditis (24 males, age 52 [23–52]). The primary endpoint was the combination of recurrent pericarditis, constrictive pericarditis, and surgery for pericardial diseases defined as pericardial events. Atrial and ventricular strain function were performed on conventional cine SSFP sequences.

**Results:**

After a median follow-up time of 16 months (interquartile range [13–24]), 12 patients with acute pericarditis reached the primary endpoint. In multivariable Cox regression analysis, LA reservoir and LA conduit strain parameters were all independent determinants of adverse pericardial diseases. Conversely, LV myocardial strain parameters did not remain an independent predictor of outcome. With receiving operating characteristics curve analysis, LA conduit and reservoir strain showed excellent predictive performance (area under the curve of 0.914 and 0.895, respectively) for outcome prediction at 12 months.

**Conclusion:**

LA reservoir and conduit mechanisms on CMR are independently associated with a higher risk of adverse pericardial events. Including atrial strain parameters in the management of acute pericarditis may improve risk stratification.

**Clinical relevance statement:**

Atrial strain could be a suitable non-invasive and non-contrast cardiovascular magnetic resonance parameter for predicting adverse pericardial complications in patients with acute pericarditis.

**Key Points:**

• *Myocardial strain is a well-validated CMR parameter for risk stratification in cardiovascular diseases.*

• *LA reservoir and conduit functions are significantly associated with adverse pericardial events.*

• *Atrial strain may serve as an additional non-contrast CMR parameter for stratifying patients with acute pericarditis.*

**Supplementary Information:**

The online version contains supplementary material available at 10.1007/s00330-024-10677-9.

## Introduction

Acute pericarditis is an inflammation of the pericardial layers, with various etiologies, including infections and autoimmune and metabolic diseases as well as radiation or iatrogenic damages [1, 2].

Although mortality related to pericardial diseases is decreasing over time, morbidity remains a persistent issue in cardiovascular healthcare [3, 4]. Accurate and timely diagnosis and management can improve patients’ outcome and prevent complications [1].

According to the current ESC (European Society of Cardiology) guidelines, the diagnosis of pericarditis primarily relies on symptoms, electrocardiogram (ECG) findings, and echocardiography features [2]. Cardiovascular magnetic resonance (CMR) is recommended for assessing myocardial involvement and for ruling out myocardial ischemia in the absence of significative coronary artery disease [2].

Indeed, CMR is a very effective non-invasive imaging modality for assessing the anatomical and morphological characteristics of the pericardial layers as well as for detecting a concomitant myocardial edema and/or scar, thanks to its high spatial resolution and tissue characterization capabilities [5–7]. In addition, CMR can provide prognostic information in patients with acute pericarditis. Conte et al demonstrated that the presence of positive late gadolinium enhancement (LGE) in the pericardial layers was significantly associated with the recurrence of pericardial events independently of clinical variables (OR 8.94, 95% CI 1.74–45.80; *p* = 0.008) [8].

The recently introduced CMR feature tracking offers a sensitive and quantitative assessment of myocardial function, enabling easy calculation of atrial and ventricular strain without the need for additional sequences and contrast media administration [6, 17]. Several studies have shown that left atrium (LA) and left ventricular (LV) strain play an increasingly important role in diagnosis, prognosis, and risk stratification of various cardiovascular diseases [9–17]. Promising diagnostic opportunities are arising through abbreviated CMR protocols that do not involve contrast media administration [13, 18–20]. The identification of predictive CMR parameters from abbreviated protocols could provide unequivocal advantages in real-life clinical practice.

To the best of our knowledge, no previous studies have investigated parameters influencing clinical outcomes in patients with acute pericarditis based on LA and LV strain mechanisms using CMR.

Therefore, the current study aimed to explore the predictive value of atrial and ventricular strain parameters derived from CMR in patients with acute pericarditis.

## Material and method

### Study population

In this retrospective, longitudinal, observational, single-center study, all patients presenting with acute pericarditis who underwent CMR between March 3rd, 2017, and December 7th, 2022, were included. We enrolled patients who met the following criteria: (1) a clinical diagnosis of the first episode of acute pericarditis according to the Position Statement of the European Society of Cardiology Heart Failure Association [2], defined by at least two of four criteria, namely pericardial chest pain, a friction rub in the pericardial region, newly observed diffuse ST-segment elevation or PR-segment depression on an electrocardiogram, and the emergence or deterioration of a pericardial effusion, and (2) had an available CMR examination within 7 days after symptom onset.

Exclusion criteria included subjects < 18 years; previous myocardial infarction; signs of myocardial involvement on CMR according to the updated Lake Louise Criteria [21]; pre-existing cardiomyopathy; a prior history of atrial fibrillation; chronic and/or recurrent pericarditis; and suspected or known prior irreversible myocardial damage.

During the initial hospitalization, clinical data and bio-humoral markers were extracted from hospital records.

Institutional Review Board approval for this study was obtained, and patient’s consent was waived because of the retrospective nature.

**Fig. 1:**
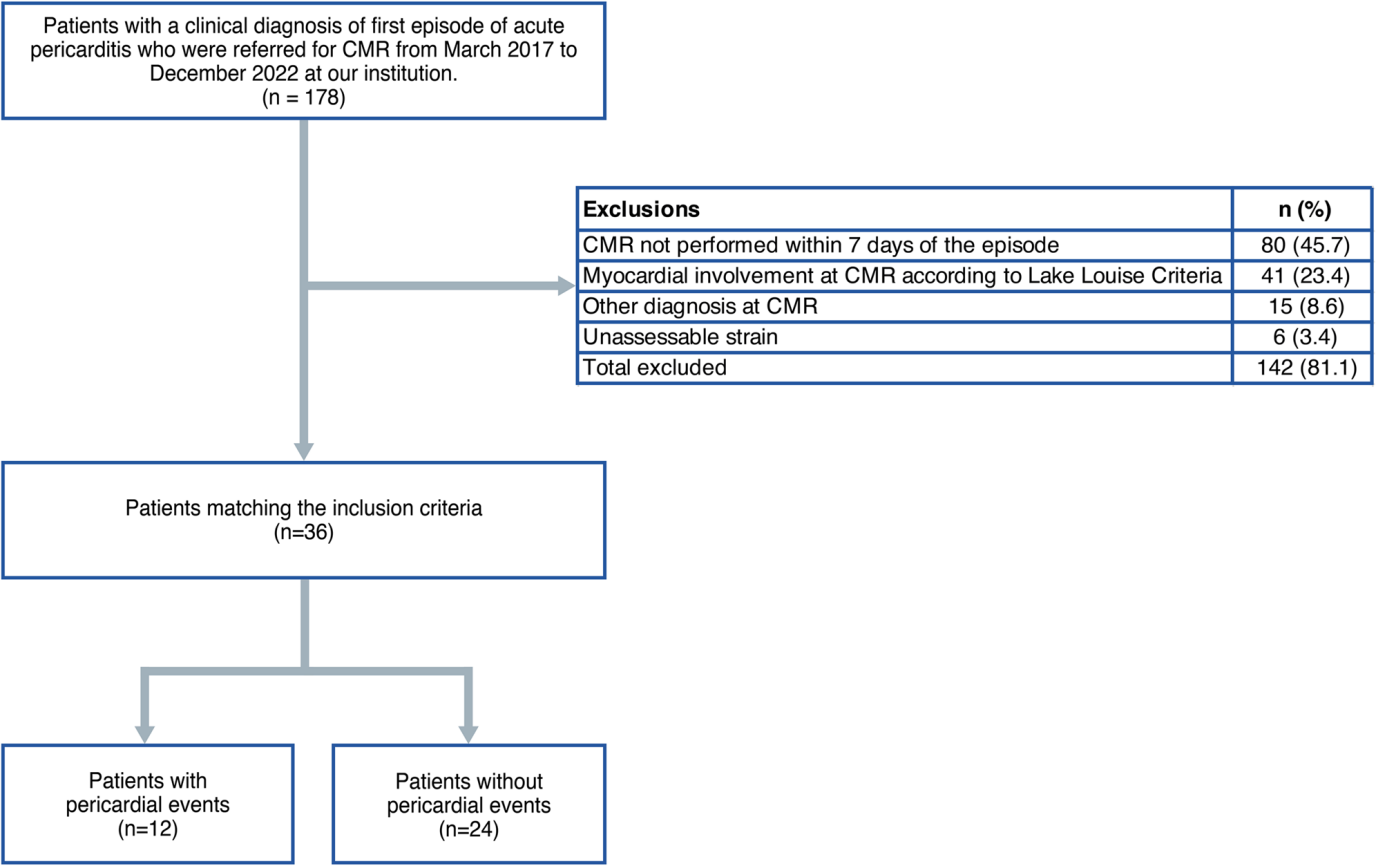
Flowchart of patients included in the study

A flowchart demonstrating the application of inclusion and exclusion criteria is provided in Fig. 1.

### CMR acquisition

CMR scans were performed at 3.7 ± 2.9 days (median = 1 day, range = 1–7 days) after admission to the hospital using a Philips Achieva dStream 1.5-T scanner system. Anterior 32-channel phased array coils were used. All cine images were acquired using a balanced steady-state free precession and retrospective gating during expiratory breath-hold maneuvers (TE, 1.7 ms; TR, 3.4 ms; flip angle, 45°; section thickness = 8 mm) in both long-axis (two-, three-, and four-chamber view) and short-axis planes with whole ventricular coverage from the base to the apex. Real-time cine images during inspiration were performed to identify the presence of biventricular interdependence, suggestive of constrictive physiology.

T1 mapping was performed in the short-axis plane in three slices (at the base, mid-ventricular, and apex, respectively) using a single-breath-hold, ECG-triggered, MOLLI sequence before contrast media injection (TE, 1.1 ms; TR, 2.5 ms; flip angle, 35°; FOV, 300 × 300 mm^2^).

LGE imaging was performed in both long- and short-axis slices 10–12 min after contrast media injection (Gadovist, Bayer Healthcare) with a dose of 0.15 mL per kg body weight using phase-sensitive inversion recovery sequences (PSIR) (TE, 2.0 ms; TR, 3.4 ms; flip angle, 20°, section thickness = 8 mm) with an inversion time determined using the Look-Locker technique.

### CMR image post-processing

We used the commercially available software system Circle CVI42 (CVI42, Circle Cardiovascular Imaging Inc.) for CMR feature tracking data analysis. Offline CMR feature tracking analyses were conducted for the evaluation of peak global longitudinal strain, global radial strain, and global circumferential strain in a 16-segment software-generated 2D model. Concerning longitudinal strain, data on myocardial strain were derived from two-, three-, and four-chamber long-axis views. Regarding radial strain and circumferential strain, data on myocardial strain was derived from apical, mid-ventricular, and basal short-axis views in all the patients. On all images, the epi- and endocardial borders were traced in end-diastole. After that, an automatic computation was triggered, during which the applied software algorithm automatically outlined the border throughout the cardiac cycle.

Similarly, CMR feature tracking analyses of atrial deformation were conducted offline. On all the acquired images, LA endocardial borders were manually traced in the long view of the cine images when the atrium was at its minimum volume. In particular, the four-, three-, and two-chamber views were used to derive LA longitudinal strain. LA appendage and pulmonary veins were excluded from segmentation.

After that, with automatic computation, the software algorithm automatically tracked the myocardial borders throughout the cardiac cycle. The quality of the tracking and contouring was visually validated and manually corrected when needed. There are three peaks in the strain curve, namely reservoir, conduit, and booster strain. Accordingly, their corresponding strain rate parameters were included. The quality of the tracking and contouring of atrial and ventricular function was visually validated and manually corrected by a radiologist with 4 years of experience in cardiac imaging.

T1 mapping values were generated offline using the same dedicated CMR software (CVI42, Circle Cardiovascular Imaging Inc.). Epi- and endocardial borders were manually traced and propagated through the image stack and manually corrected when needed.

For interobserver analysis, an additional blinded observer (M.P., with 6 years of experience in cardiovascular imaging) independently conducted strain analysis on a randomly selected set of 20 patients.

Pericardial effusion quantification was obtained by directly delineating it on cine-CMR in the end-diastolic short-axis view, measuring its maximal extent [22].

The presence of LGE in the pericardium was evaluated using both qualitative and semiquantitative methods, as previously described [23]. In brief, LGE in the pericardium was categorized as follows: none (no apparent LGE visible), mild (subtle LGE in the pericardium with signal intensity lower than that of the ventricular blood pool), moderate (clear enhancement resembling the ventricular blood pool), or severe (significant and visually prominent LGE in the pericardium with signal intensity higher than that of the ventricular blood pool) [23].

### Study end points

All patients were followed up by clinical visits after the CMR examinations, and hospital records were screened for clinical events. The endpoint was the composite of pericardial complications (defined as recurrent pericarditis, constrictive pericarditis, and surgery for pericardial disease) according to the ESC guidelines [2]. The diagnosis of recurrent pericarditis is established when a patient meets all criteria: (1) a documented initial episode of acute pericarditis, (2) a symptom-free interval lasting at least 4 weeks, and (3) a subsequent recurrence determined by the criteria for acute pericarditis [2].

### Statistical analysis

Continuous variables were presented as mean (standard deviation [SD]), while categorical variables were expressed as frequency (%). Comparisons of continuous variables were conducted through Welch’s *t*-test, with Kolmogorov–Smirnov tests employed to assess the normality of residuals. Categorical variables were analyzed using the chi-square test or Fisher’s exact test, as appropriate. Univariable analysis was performed using Cox proportional hazard (PH) regression to identify independent predictors of pericardial events. Atrial and ventricular strain predictors that demonstrated statistical significance (*p* < 0.05) during univariable analysis were subjected to further examination through multivariable Cox regression, adjusting for all factors that were statistically significant in the univariable analysis. The cut-offs for significant LA strain predictors were determined by analyzing the relationships between predictor’s values and hazard ratios (HR) determined through Cox PH regression. These cut-offs were set at the point where the HR intersected with 1, indicating a neutral association with the outcome. Subsequently, these cut-offs were applied to stratify patients into low- and high-risk groups. The survival function of patients in each subgroup was then explored using Kaplan–Meier curves. For time-dependent ROC analysis at *t* = 12 months, we utilized the R package timeROC [24]. The computation of areas under the curve was executed using the trapezoidal rule approximation method. Pointwise confidence intervals were derived from the asymptotic normality of time-dependent areas under the curve estimators, employing inverse probability of censoring weights computed from a Kaplan–Meier estimator [25].

Statistical differences in time-dependent areas under the curve at *t* = 12 months were assessed. To account for the influence of confounding factors, the survival probability of continuous covariates that remained significant during multivariable analysis was calculated as a probability area using g-computation [26].

All statistical tests were two-sided and a *p*-value < 0.05 was considered statistically significant. All statistical analyses were performed using R Statistical Software (v4.2.2; R Core Team 2022).

## Results

### Patient population

During the inclusion period, a total of 36 patients with acute pericarditis were enrolled after the application of inclusion and exclusion criteria (Fig. 1). Baseline characteristics of patients are shown in Table 1.

**Table 1:** Baseline and CMR characteristic of patients with and without adverse pericardial events

Variable	Overall, *N* = 36^1^	Event, *N* = 12^1^	No event, *N* = 24^1^	*p*-value^2^
Gender (male), *n* (%)	26 (72%)	9 (75%)	17 (71%)	> 0.99
Age, years	48 (23)	61 (23)	41 (20)	**0.020**
Height, cm	169 (8)	171 (10)	169 (7)	0.53
Weight, kg	69 (13)	75 (12)	66 (12)	**0.033**
BMI, kg/m^2^	24 (4.2)	26 (4.9)	23 (3.6)	0.077
Hypertension, *n* (%)	17 (47%)	7 (58%)	10 (42%)	0.35
Dyslipidemia, *n* (%)	7 (19%)	3 (25%)	4 (17%)	0.66
Obesity,* n* (%)	5 (14%)	3 (25%)	2 (8.3%)	0.31
Current or previous smoking, *n* (%)	8 (22%)	4 (33%)	4 (17%)	0.40
Diabetes mellitus, *n* (%)	4 (11%)	3 (25%)	1 (4.2%)	0.10
Family history of coronary disease, *n* (%)	9 (25%)	4 (33%)	5 (21%)	0.44
Chest pain, *n* (%)	24 (67%)	8 (67%)	16 (67%)	> 0.99
Heart failure,* n* (%)	3 (8.3%)	2 (17%)	1 (4.2%)	0.25
Arrhythmias, *n* (%)	1 (2.8%)	1 (8.3%)	0 (0%)	0.33
Leukocytosis, *n* (%)	12 (33%)	4 (33%)	8 (33%)	> 0.99
CRP, *n* (%)	29 (81%)	8 (67%)	21 (88%)	0.19
Erythrocyte sedimentation rate, *n* (%)	11 (31%)	6 (50%)	5 (21%)	0.12
Fever, *n* (%)	14 (39%)	6 (50%)	8 (33%)	0.47
Troponin, *n* (%)	9 (25%)	2 (17%)	7 (29%)	0.69
Subacute course, *n* (%)	10 (28%)	3 (25%)	7 (29%)	> 0.99
Respond to non-steroidal anti-inflammatory drugs, *n* (%)	19 (53%)	4 (33%)	15 (63%)	0.10
Pericardial thickness, mm	3.22 (2.67)	3.67 (2.96)	3.00 (2.55)	0.51
Pericardial effusion, *n* (%)	16 (44%)	7 (58%)	9 (38%)	0.24
Pericardial effusion (thickness), mm	6 (10)	11 (13)	3 (7)	0.078
Reservoir, %	29 (12)	19 (9)	33 (11)	** < 0.001**
Reservoir rate, %	1.34 (0.59)	0.98 (0.51)	1.53 (0.55)	**0.007**
Conduit, %	17 (10)	9 (5)	21 (9)	** < 0.001**
Conduit rate, %	− 1.72 (0.87)	− 1.25 (0.83)	− 1.95 (0.81)	**0.025**
Booster, %	13 (6)	10 (6)	14 (6)	**0.049**
Booster rate, %	− 1.64 (0.64)	− 1.25 (0.57)	− 1.83 (0.59)	**0.009**
LV ejection fraction, %	57 (7)	58 (7)	56 (8)	0.45
LV mass index, g	104 (27)	105 (25)	103 (28)	0.87
BSA-indexed LV end-diastolic volume, mL/m^2^	86 (25)	75 (27)	91 (23)	0.084
BSA-indexed LV end-systolic volume, mL/m^2^	37 (16)	30 (15)	40 (17)	0.074
BSA-indexed LV stroke volume, mL/m^2^	49 (11)	45 (16)	51 (8)	0.19
BSA-indexed LV mass, g/m^2^	59 (14)	58 (15)	60 (14)	0.73
RV ejection fraction, %	56.3 (6.0)	56.5 (6.4)	56.2 (5.9)	0.90
BSA-indexed RV end-diastolic volume, mL/m^2^	99 (137)	70 (20)	113 (166)	0.22
BSA-indexed RV end-systolic volume, mL/m^2^	35 (11)	32 (14)	36 (10)	0.37
BSA-indexed RV stroke volume, mL/m^2^	43 (9)	39 (11)	45 (8)	0.083
LV strain, %				
Basal radial	25 (9)	27 (10)	24 (8)	0.45
Basal circumferential	− 15.2 (3.9)	− 15.8 (4.5)	− 14.9 (3.6)	0.55
Basal longitudinal	− 13.6 (3.3)	− 13.8 (2.8)	− 13.6 (3.5)	0.83
Mid radial	23 (12)	28 (9)	21 (12)	0.082
Mid circumferential	− 14.7 (6.2)	− 17.4 (3.3)	− 13.3 (6.9)	**0.024**
Mid longitudinal	− 14.94 (3.22)	− 14.73 (2.43)	− 15.04 (3.59)	0.76
Apical radial	36 (19)	41 (13)	34 (21)	0.24
Apical circumferential	− 18.4 (7.2)	− 20.9 (3.9)	− 17.2 (8.1)	0.073
Apical longitudinal	− 14.0 (3.8)	− 13.1 (3.6)	− 14.5 (3.9)	0.29
Global radial	26 (10)	30 (10)	25 (10)	0.18
Global circumferential	− 15.4 (5.5)	− 17.1 (4.3)	− 14.6 (6.0)	0.16
Global longitudinal	− 14.1 (2.9)	− 13.9 (2.5)	− 14.3 (3.2)	0.65
LGE pericardial enhancement, *n* (%)	21 (58%)	8 (67%)	13 (54%)	0.47
LGE grading, *n* (%)				0.068
No pericardial LGE	13 (36%)	4 (33%)	9 (38%)	
Mild pericardial LGE	5 (14%)	0 (0%)	5 (21%)	
Moderate pericardial LGE	11 (31%)	3 (25%)	8 (33%)	
Severe pericardial LGE	7 (19%)	5 (42%)	2 (8.3%)	
T2 STIR, n (%)	16 (44%)	7 (58%)	9 (37%)	0.14
T1 mapping, ms	1035 (154)	1057 (55)	1025 (185)	0.44

Abbreviations: *BSA*, body surface area; *CRP*, C-reactive protein; *EDV*, end-diastolic volume; *ESV*, end-systolic volume; *LGE*, late gadolinium enhancement; *LV*, left ventricle; *STIR*, short tau inversion recovery; *SV*, stroke volume; *RV*, right ventricle

^1^Mean (SD) or frequency (%)

^2^Fisher’s exact test; Welch two-sample *t*-test; Pearson’s chi-squared test

Among the patients enrolled, 30 had idiopathic pericarditis and 6 had non-infectious causes of pericarditis, including 4 with connective tissue diseases and 2 with a history of previous radiotherapy.

**Fig. 2:**
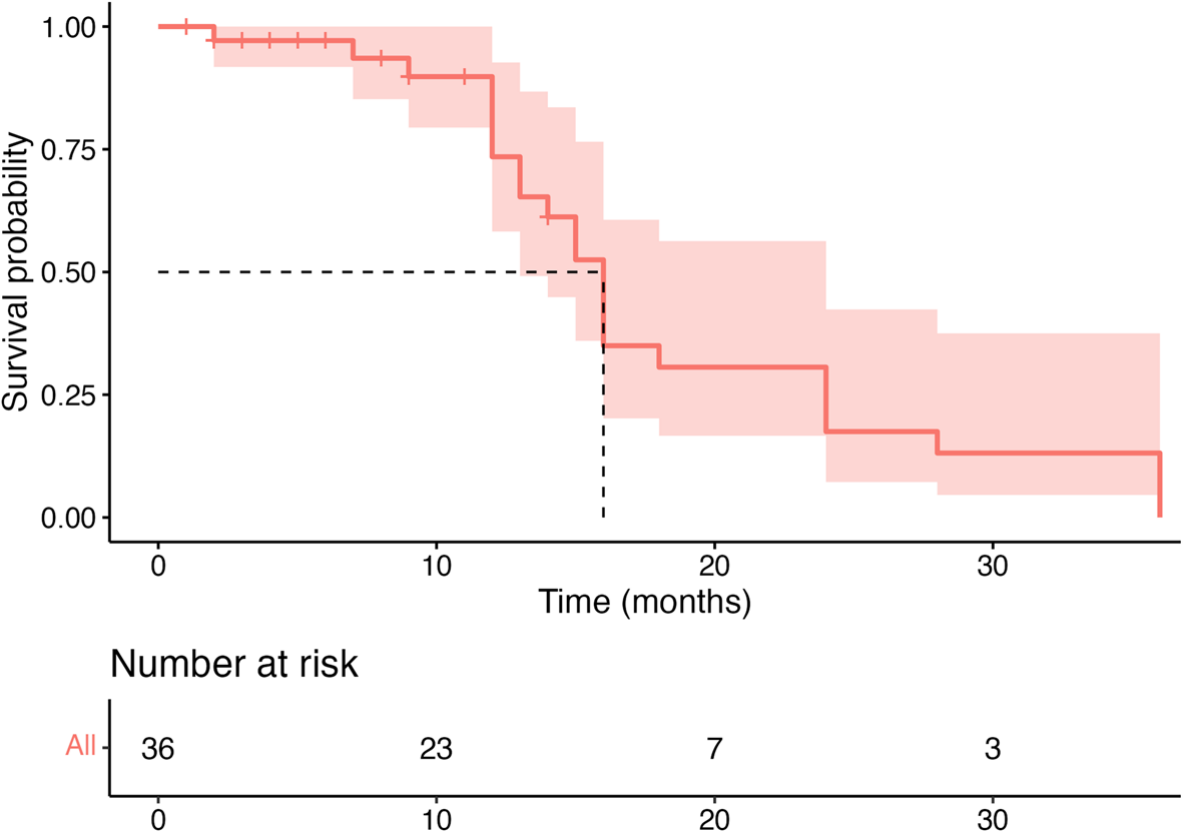
Pericardial event-free survival during follow-up. Kaplan–Meier curve showing the probability of pericardial event-free survival during follow-up. The median survival is indicated with dashed lines. The table at the bottom shows the population at risk at different time points during follow-up

During a median follow-up of 16 months (IQR [13–24]; Fig. 2), 12 patients (33%) had a pericardial event (age 61 [38–84]), including 6 recurrences, 4 chronic pericarditis, and 2 pericardial surgeries; 24 patients (67%) completed the follow-up period event-free (age 41 [21–61]).

The patients’ mean age was 48 (25–71) with 24 males and 9 females. Patients who experienced pericardial events at follow-up were older (age 61 [38–84] vs. age 41 [21–61], *p* = 0.02) with no significant differences between the two groups in terms of cardiovascular risk factors at baseline.

Leukocytosis was observed in 12 (33%) patients, whereas high c-reactive protein and high erythrocyte sedimentation rate presented in 29 (81%) and 11 (31%), respectively. No differences were observed in laboratory data between patients with vs without pericardial events.

Among the CMR features, the presence of pericardial effusion and positive LGE and T2-STIR on the pericardium did not show a significant difference between the two enrolled groups. Conversely, all LA strain and strain rate parameters as well as LV mid circumferential strain were impaired among subjects with pericardial events at follow-up (Table 1).

### Associations of ventricular and atrial strain measures with pericardial events

**Fig. 3:**
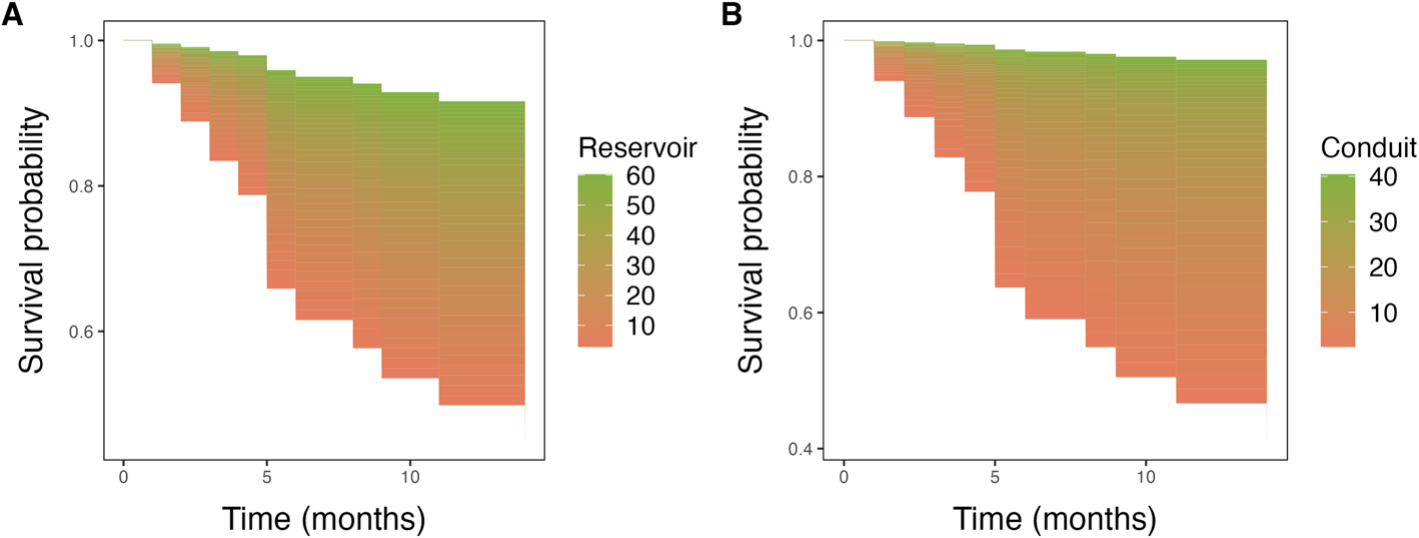
**A**, **B** Survival probability by values of prognosticators during follow-up. 3D survival areas for left atrial (LA) reservoir and LA conduit, illustrating the survival probability (*y*-axis) at various time points during follow-up (*x*-axis) across different prognosticator values (color-coded). For instance, panel **A** demonstrates higher survival probability within 12 months associated with higher LA reservoir values

**Fig. 4:**
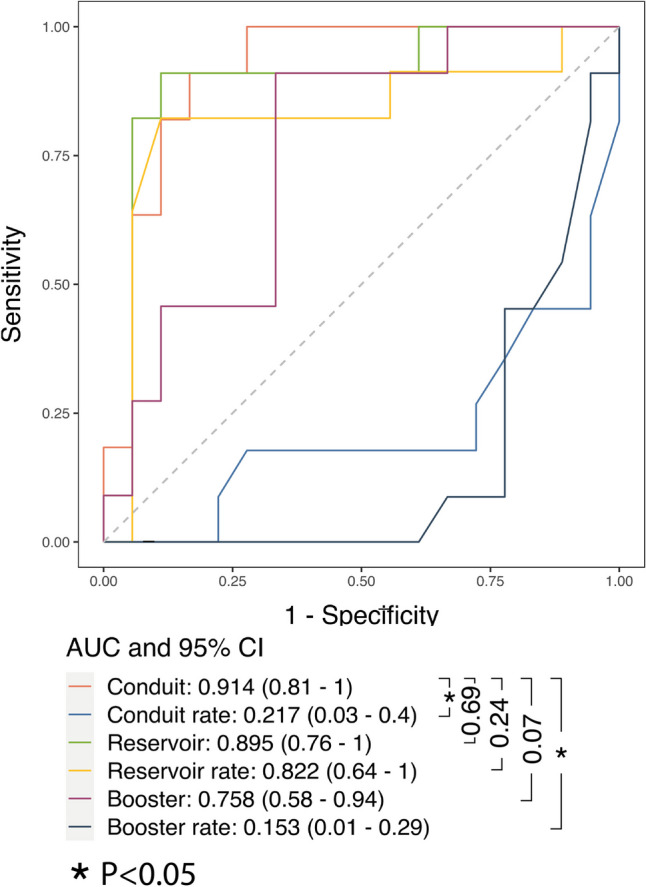
Time-dependent ROC curves at *t* = 12 months. This graph visually evaluates the performance of survival models adjusted for age, diabetes, pericardial effusion (thickness), and BSA-indexed left ventricle stroke volume in predicting adverse pericardial events within a 12-month timeframe. It reports the areas under the curve along with their corresponding 95% confidence intervals. Vertical whiskers denote statistical comparisons of the area under the curve values

Univariable analysis revealed that weight, the presence of diabetes mellitus, pericardial effusion thickness, and higher conduit rate and booster rate values were significantly associated with a higher risk for adverse pericardial events during follow-up. Conversely, higher LA reservoir, LA reservoir rate, LA conduit, LA booster, and BSA-indexed LV stroke volume (SV) were significantly associated with lower risk of pericardial events during follow-up (Table 2). Further multivariable analysis revealed that LA reservoir and LA conduit were statistically significant independent predictors of adverse pericardial events after factoring out the influence of age, diabetes mellitus, LV SV/BSA, and pericardial effusion thickness (Table 3). In particular, higher values of LA reservoir and LA conduit were associated with a lower risk of pericardial events at follow-up (Fig. 3). Cut-offs identified by analyzing the relationships between LA reservoir and LA conduit with hazard ratios by a Cox PH regression (28% and 17%, respectively) allowed for a statistically significant stratification of patients into low- and high-risk groups as shown in Supplemental Fig. 1. Time-dependent ROC analysis revealed excellent predictive performance of the adjusted models of LA reservoir and LA conduit, with respective areas under curve of 0.895 (95% confidence interval [CI], 0.76–1.0) and 0.914 (95% CI, 0.81–1.0), in predicting adverse pericardial events within 12 months. Notably, both models significantly outperformed the conduit rate–based and booster rate–based models in outcome prediction at 12 months (0.895 and 0.914 vs. 0.217 (95% CI, 0.03–0.40) and 0.153 (95% CI, 0.01–0.29), all *p* < 0.05) (Fig. 4).

**Table 2:** Univariable Cox proportional hazards regression analysis of clinical and CMR characteristics for prediction of adverse pericardial events

Variable	Hazard ratio (95% CI)	*p*-value
Gender	1.2 (0.31–4.3)	0.83
Age	1 (1–1.1)	**0.024**
Height	1 (0.95–1.1)	0.51
Weight	1.1 (1–1.1)	**0.031**
Hypertension	1.8 (0.56–5.6)	0.33
Dyslipidemia	1.7 (0.45–6.3)	0.44
Obesity	2.2 (0.6–8.2)	0.23
Current or previous smoking	1.5 (0.45–4.9)	0.52
Diabetes mellitus	4 (1–15)	**0.044**
Family history of coronary disease	1.6 (0.48–5.3)	0.45
Chest pain	1 (0.3–3.3)	0.99
Heart failure	2.5 (0.54–12)	0.24
Arrhythmias	3.2 (0.41–26)	0.27
Leukocytosis	1.1 (0.34–3.8)	0.84
CRP	0.43 (0.13–1.4)	0.17
Erythrocyte sedimentation rate	2.6 (0.84–8.2)	0.097
Fever	2.2 (0.69–6.8)	0.18
Troponin	0.64 (0.14–2.9)	0.57
Subacute course	0.98 (0.27–3.6)	0.98
Respond to non-steroidal anti-inflammatory drugs	0.35 (0.1–1.2)	0.092
Pericardial thickness	1.1 (0.85–1.3)	0.6
Pericardial effusion	1.9 (0.6–6.1)	0.28
Pericardial effusion (thickness)	1 (1–1.1)	**0.029**
Reservoir	0.92 (0.88–0.97)	**0.0012**
Reservoir rate	0.29 (0.11–0.77)	**0.013**
Conduit	0.85 (0.78–0.94)	**0.00087**
Conduit rate	2.5 (1.1–5.9)	**0.034**
Booster	0.9 (0.81–0.99)	**0.036**
Booster rate	3 (1.3–6.9)	**0.011**
LV ejection fraction	1 (0.94–1.1)	0.61
LV mass index	1 (0.98–1)	0.74
BSA-indexed LV end-diastolic volume	0.98 (0.96–1)	0.068
BSA-indexed LV end-systolic volume	0.96 (0.92–1)	0.097
BSA-indexed LV stroke volume	0.95 (0.9–1)	**0.049**
BSA-indexed LV mass	1 (0.96–1)	0.83
RV ejection fraction	1 (0.91–1.1)	0.95
BSA-indexed RV end-diastolic volume	0.97 (0.94–1)	0.13
BSA-indexed RV end-systolic volume	0.97 (0.9–1)	0.33
BSA-indexed RV stroke volume	0.94 (0.88–1)	0.051
LV strain		
Basal radial	1 (0.96–1.1)	0.41
Basal circumferential	0.95 (0.81–1.1)	0.52
Basal longitudinal	0.99 (0.83–1.2)	0.88
Mid radial	1 (0.98–1.1)	0.16
Mid circumferential	0.87 (0.74–1)	0.084
Mid longitudinal	1 (0.88–1.2)	0.62
Apical radial	1 (0.98–1)	0.43
Apical circumferential	0.94 (0.85–1)	0.2
Apical longitudinal	1.1 (0.97–1.3)	0.12
Global radial	1 (0.98–1.1)	0.22
Global circumferential	0.93 (0.81–1.1)	0.26
Global longitudinal	1.1 (0.88–1.3)	0.5
LGE pericardial enhancement	1.5 (0.44–4.9)	0.53
LGE grading	1.5 (0.88–2.5)	0.14
T2 STIR	2 (0.85–4.9)	0.11
T1 mapping	1 (1–1)	0.61

*BSA*, body surface area; *CRP*, C-reactive protein; *EDV*, end-diastolic volume; *ESV*, end-systolic volume; *LGE*, late gadolinium enhancement; *LV*, left ventricle; *STIR*, short tau inversion recovery; *SV*, stroke volume; *RV*, right ventricle

**Table 3:** Multivariable Cox proportional hazards regression analysis. All atrial strain parameters were adjusted for factors that were statistically significant in the univariable analysis

	Multivariable analysis	
	Hazard ratio (95% CI)	*p*-value
Reservoir		
** Reservoir**	**0.94 (0.89–1.00)**	**0.038**
Age	1.01 (0.96–1.06)	0.758
Diabetes mellitus	1.89 (0.28–12.75)	0.514
Pericardial effusion (thickness)	1.01 (0.95–1.07)	0.699
LV SV/BSA	0.98 (0.91–1.05)	0.504
Reservoir rate		
Reservoir rate	0.43 (0.13–1.36)	0.150
Age	1.01 (0.97–1.06)	0.615
Diabetes mellitus	1.95 (0.30–12.67)	0.486
Pericardial effusion (thickness)	1.02 (0.97–1.08)	0.473
LV SV/BSA	0.97 (0.91–1.04)	0.437
Conduit		
** Conduit**	**0.86 (0.76–0.97)**	**0.017**
Age	0.99 (0.93–1.04)	0.634
Diabetes mellitus	2.10 (0.30–14.63)	0.453
Pericardial effusion (thickness)	1.00 (0.95–1.06)	0.930
LV SV/BSA	0.96 (0.89–1.03)	0.275
Conduit rate		
Conduit rate	1.30 (0.41–4.08)	0.657
Age	1.02 (0.97–1.07)	0.538
Diabetes mellitus	1.99 (0.31–12.90)	0.472
Pericardial effusion (thickness)	1.02 (0.97–1.08)	0.354
LV SV/BSA	0.98 (0.91–1.05)	0.491
Booster		
Booster	0.92 (0.83–1.02)	0.109
Age	1.02 (0.98–1.07)	0.299
Diabetes mellitus	1.86 (0.28–12.19)	0.518
Pericardial effusion (thickness)	1.02 (0.96–1.08)	0.570
LV SV/BSA	0.98 (0.92–1.05)	0.607
Booster rate		
Booster rate	2.23 (0.97–5.12)	0.059
Age	1.02 (0.97–1.07)	0.492
Diabetes mellitus	2.09 (0.30–14.69)	0.459
Pericardial effusion (thickness)	1.02 (0.96–1.08)	0.475
LV SV/BSA	0.98 (0.91–1.05)	0.552

*CI*, indicates confidence interval; *LV SV*, left ventricular stroke volume; *BSA*, body surface area

Good interobserver agreement was observed for both atrial and ventricular strain parameters, with intraclass correlation coefficients ranging from 0.821 to 0.881.

## Discussion

The current study evaluated the prognostic impact of LA and LV myocardial strain in patients with acute pericarditis. Our results indicate that both reservoir and conduit strain parameters independently predict adverse pericardial events.

Acute pericarditis is characterized by inflammation affecting the pericardium. From a histopathological point of view, the pericardial sac responds to injuries by producing exudate, which includes a mixture of fluid, fibrin, and cells [27]. Focal and/or diffuse fibrin deposits on the pericardial surface are commonly encountered on histological specimens [27]. These pericardial reactions to injury may influence physiological myocardial contraction. Human and animal studies have demonstrated that the pericardium primarily affects diastolic function [28], potentially leading to compensatory strain changes aimed at preserving LV systolic performance [29].

The normal pericardium plays a significant role in preserving the Frank-Starling mechanism while interacting with ventricular filling [28]. Acute pericarditis alters the viscoelastic properties of the pericardium, resulting in changes in pericardial distensibility and a subsequent increase in pericardial restraint, influencing ventricular filling [30].

The LA is an active cardiac chamber that plays a central role in modulating LV filling through its distinct phases [12, 31, 32], namely (1) functioning as a reservoir for blood from pulmonary veins during LV systole, (2) a conduit for blood transiting from the pulmonary veins to the LV during the early diastole, and (3) a booster pump that acts as an active contractile chamber to enhance LV filling during late diastole [14]. LA strain has, therefore, shown to be an effective CMR parameter of diastolic function [33].

In addition, LA strain parameters have shown to influence the outcomes of various cardiovascular diseases [14].

However, little is known about the impact and prognosis of LA mechanism in acute pericarditis. Our results suggested an impairment in atrial function in patients who experienced pericardial events. The prognostic value of LA reservoir and conduit strain parameters may be related to their sensibility to altered LV filling. The integration of faster and more cost-effective CMR protocols in clinical practice presents undeniable advantages. This strategy not only improves the accessibility of CMR examinations but also extends their availability to a broader patient population, including those unable to receive contrast agents or tolerate lengthy procedures. Consequently, there is a growing motivation to explore alternative markers that do not necessitate contrast administration while still effectively predicting outcomes in pericarditis patients.

Of interest, positive LGE in the pericardium did not show an association with adverse pericardial events, contrary to previous reports [8]. A potential explanation of this discrepancy could be related to difference in enrolled cohorts. We excluded patients with a history of prior myocardial infarction and those exhibiting signs of myocardial involvement meeting the Lake Louise Criteria. Notably, pericardial involvement has been established as an independent prognostic predictor for worse cardiac outcomes in patient with acute myocarditis [34] and acute myocardial infarction [35].

Acute pericarditis represents a significant cause of morbidity, with potential complications such as cardiac tamponade, constrictive pericarditis, and episodes of recurrence [36]. Early recognition of patients at high risk of pericardial events is useful in clinical practice, allowing more tailored therapy and improving outcomes.

This study has certain limitations. Firstly, due to its retrospective design, some clinical and laboratory data was not available for analysis in every patient. Secondly, the relatively modest sample size, coupled with a limited number of events, may introduce overfitting risk in our multivariable analysis. Therefore, information on the incremental prognostic value of the models is limited. It is also noteworthy that the limited timeframe of follow-up, coupled with a relatively high percentage of patients with a follow-up less than or equal to 12 months (25%), may have influenced our findings, which could have been more robust statistically with a larger number of patients undergoing a longer follow-up. Further studies with a larger sample size and a higher number of events are warranted to validate and strengthen these findings. Furthermore, the absence of a dedicated validation set warrants careful consideration when extrapolating their generalizability to a broader population. Although our study yielded promising results, it is essential to conduct further prospective trials involving a larger patient cohort to validate our findings.

## Conclusion

LA reservoir and conduit mechanisms are independently associated with a higher risk of adverse pericardial events. The LA strain may serve as an additional non-contrast CMR parameter in stratifying patients with acute pericarditis. The current findings, if confirmed in larger prospective multicenter studies, could help tailor treatments and improve risk stratification in acute pericarditis patients.

### Supplementary Information

Below is the link to the electronic supplementary material.

Supplementary file1 (PDF 338 KB)

## Abbreviations

CMR: Cardiovascular magnetic resonance
ECG: Electrocardiogram
ESC: European Society of Cardiology
LA: Left atrium
LGE: Late gadolinium enhancement
LV: Left ventricle

## Declarations

### Guarantor

The scientific guarantor of this publication is the corresponding author.

### Statistics and biometry

One of the authors has significant statistical expertise.

### Study subjects or cohorts overlap

No study subject or cohort overlap has been reported.

### Methodology

• retrospective

• diagnostic or prognostic study

• performed at one institution
