# Supplementary material for: Atrial and ventricular strain using cardiovascular magnetic resonance in the prediction of outcomes of pericarditis patients: a pilot study
Source: Eur Radiol. 2024 Mar 11;34(9):5724–35. doi: 10.1007/s00330-024-10677-9 (PMC11364562; doi:10.1007/s00330-024-10677-9)
Supplement: Supplementary file 1 — Supplementary file1 (PDF 338 KB) [file 330_2024_10677_MOESM1_ESM.pdf]

Supplemental Appendix for manuscript:

Atrial and ventricular strain using cardiovascular magnetic resonance in the prediction of outcomes of pericarditis patients: A pilot study.

Eur Radiol (2024) Cau R, Pisu F, Muscogiuri G et al

Supplemental Figures Legends

Supplemental Figure 1. Relationship between Left atrium (LA) reservoir and LA conduit strain and the occurrence of pericardial events during follow-up. A and C) Values of LA reservoir and LA conduit and corresponding hazard ratios by Cox proportional hazard survival analysis. Cut-offs were established at the point where the hazard ratio intersected with 1, allowing for a meaningful distinction in risk categories. B and D) Kaplan-Meier curves exploring the survival function of patients after stratification into low- and high-risk groups based on the identified cut-offs for LA reservoir and LA conduit. The p-values for the log-likelihood test, comparing the two survival curves, are provided in the bottom left corner of each panel. Additionally, tables at the bottom of the figure present the number of patients at risk for the respective subgroups determined by the application of these cut-offs.

Supplemental Figures

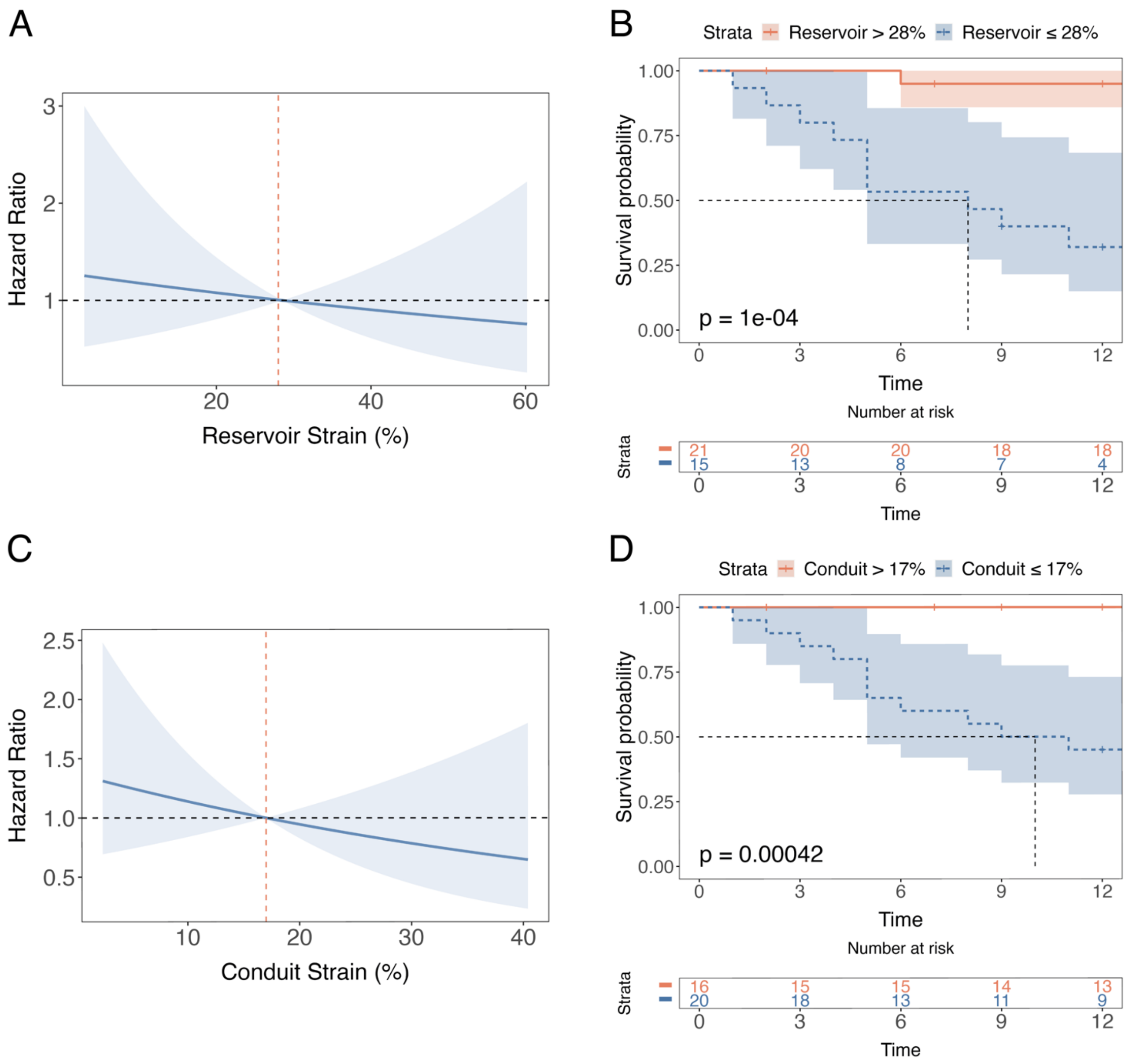

Supplemental Figure 1.
